# Orai1 downregulation causes proliferation reduction and cell cycle arrest via inactivation of the Ras-NF-κB signaling pathway in osteoblasts

**DOI:** 10.1186/s12891-022-05311-y

**Published:** 2022-04-11

**Authors:** Yunshan Guo, Jinzhu Fan, Shuguang Liu, Dingjun Hao

**Affiliations:** 1 Department of spinal surgery, Hong Hui Hospital, Xi’an Jiao Tong University, Xi’an, Shaanxi 710054 People’s Republic of China; 2 Department of bone microsurgery, Honghui Hospital, Xi’an Jiaotong University, Xi’an, Shaanxi 710054 People’s Republic of China; 3 Department of Joint Surgery, Honghui Hospital, Xi’an Jiaotong University, Xi’an, Shaanxi 710054 People’s Republic of China

**Keywords:** Orai1, Ras, NF-κB, Cyclin D1, Proliferation, Osteoblasts

## Abstract

**Background:**

The purpose of this study was to determine the role of Orai1 in the regulation of the proliferation and cell cycle of osteoblasts.

**Methods:**

The expression of Orai1 was inhibited by Orai1 small interfering RNA (siRNA) in MC3T3-E1 cells. Following Orai1 downregulation, cell proliferation and cell cycle were examined. Furthermore, the expression of cyclin D1, cyclin E, CDK4, and CDK6 was analyzed. The activity of the Ras-NF-κB signaling pathway was investigated to identify the role of Orai1 in the regulation of osteoblast proliferation.

**Results:**

Orai1 was successfully downregulated in MC3T3-E1 cells by the Orai1 siRNA transfection (*p* < 0.05). We found that MC3T3-E1 cell proliferation was decreased, and the cell cycle was arrested by Orai1 downregulation (*p* < 0.05). Additionally, the expression of cyclin D1 was decreased by Orai1 downregulation (*p* < 0.05), as was the activity of the Ras-NF-κB signaling pathway (*p* < 0.05). Orai1 siRNA did not further reduce cell proliferation, the proportion of cells in the S phase, and cyclin D1 expression after chemical blockage of the Ras signaling pathway in MC3T3-E1 cells (*p* > 0.05).

**Conclusions:**

The results reveal that Orai1 downregulation may reduce cyclin D1 expression by inactivating the Ras-NF-κB signaling pathway thus blocking osteoblast proliferation and cell cycle.

**Supplementary Information:**

The online version contains supplementary material available at 10.1186/s12891-022-05311-y.

## Introduction

Osteoporosis is a common degenerative disease of the skeletal system and seriously affects the health of the elderly. The inhibition of osteoblast proliferation leads to the onset and development of osteoporosis [1–5]. Therefore, a study on the mechanism regulating osteoblast proliferation will lead to a new biological treatment of clinical osteoporosis. The calcium ion is an important secondary messenger of cells and is involved in the regulation of cell proliferation, differentiation, migration, and cytokine secretion [6, 7]. For excitable cells, a pathway launched by a voltage-dependent calcium channel located on the plasma membrane is the major cascade that mediates the influx of extracellular calcium into the cytoplasm. A voltage-dependent calcium channel is not present in most nonexcitable cells, and recent research indicates that a pathway triggered by a calcium release-activated calcium channel (CRAC) is the primary cascade that mediates extracellular calcium influx into the cytoplasm of nonexcitatory cells. Orai1 is the key component of a calcium release-activated calcium channel (CRAC). Orai1 mainly comprises four transeptal segments and is a quaternary transmembrane protein. It is located on the plasma membrane, and its C and N termini are both located in the cell. The first segment of Orai1 is rich in proline and arginine, and the third transeptal segment is rich in glutamate and can selectively bind and transport calcium ions. When extracellular stimuli cause depletion of endoplasmic-reticulum calcium, stromal interaction molecule 1 (STIM1), located on the endoplasmic omentum, can induce Orai1 to form a calcium channel that promotes calcium ion influx and activates calcium signaling cascades to complete various cellular physiological functions [8, 9]. Previous studies have revealed that Orai1 may be a target for the treatment or prevention of the bone loss caused by osteoclasts in the RAW cell line. Orai1 knockdown reduces store-operated calcium entry (SOCE) and inhibits RANKL-induced osteoclastogenesis of RAW264.7 cells, a murine monocyte/macrophage cell line, by suppressing the induction of NFATc1 [10]. Osteoblast differentiation and function are affected by Orai1 deficiency. Deletion of the Orai1 gene can lead to a phenotype of osteoporosis, e.g., decreased bone density in mice, suggesting that Orai1 participates in the pathogenesis of osteoporosis [11–13]. Nonetheless, to the best of our knowledge, there are no reports about Orai1 involvement in the proliferation of osteoblast. Our study indicates that Orai1 downregulation leads to a slowdown of osteoblast proliferation and to cell cycle arrest. The Orai1 downregulation inactivates the Ras-NF-κB signaling pathway to reduce the expression of cyclin D1 thus decreasing the proliferation of osteoblasts. The purpose of this study was to uncover new avenues for the treatment of osteoporosis.

## Material and methods

### Cell culture

Mouse calvaria osteoblasts, MC3T3-E1, were purchased from the American Type Culture Collection (ATCC) and cultured at 37 °C in α-Minimum Essential Medium supplemented with 10% of fetal bovine serum (Gibco, NY, USA). The culture medium was refreshed every three days thereafter.

### Gene silencing

The sense sequence for Orai1 small interfering RNA (siRNA) was 5`-CGTGCACAATCTCAACTCG-3`. Orai1 siRNA was dissolved in the serum-free α-Minimum Essential Medium with the Lipofectamine 2000 reagent for 20 min. Then, 40 nM Orai1 siRNA (or control siRNA) was added into 6-well plates containing MC3T3-E1 cells. After transfeced for 6 h, the transfection solution was discarded and the cells were cultured in α-Minimum Essential Medium with 10% FBS for further analysis.

### Immunofluorescence analysis

Approximately 10^4^ cells per well were added into a 24-well cell culture plate. After the cells were cultured for 4 h, the cells were fixed with a 4% methanol-free formaldehyde solution and rinsed with PBS twice. Then the Orai1 antibody (Invitrogen, CA, USA) were diluted 1:500 in Tris-buffered saline and incubated with the cells at 37 °C for 60 min. To detect Orai1, the cells were incubated for 40 min with a FITC-conjugated IgG antibody (Pierce, Illinois, USA) diluted 1:800 in TBST. Finally, the slides were incubated with 4,6-diamidino-2-phenylindole (DAPI) (Pierce, Illinois, USA) to stain nuclei for 20 min. The cells were immediately analyzed under a fluorescence microscope with a standard fluorescein filter set (Olympus, Japan) to examine green fluorescence of Orai1 at 520 nm and blue fluorescence of DAPI-stained nuclei at 460 nm. Each experiment was carried out in triplicate and repeated three times independently.

### Western blot analysis

The pretreated cells were lysed, and the total protein concentration was determined using the BCA Protein Assay Kit (Pierce, Illinois, USA). Equal quantities of cell lysate samples were mixed with loading buffer and then separated by sodium dodecyl sulphate-polyacrylamide gel electrophoresis in a 10% gel for 90 min. After that, the proteins were electrophoretically transferred from the gel onto a polyvinylidene fluoride microporous (PVDF) membrane for 60 min in a semidry chamber. Anti-mouse Orai1 antibodies (#PA5–109270) and GAPDH (#A21994) antibodies were purchased from Invitrogen (Carlsbad, CA, USA). The following anti-mouse antibodies were purchased from Cell Signaling Technology (Beverly, MA, USA): cyclin D1 antibodies (#55506), cyclin E antibodies (#20808), Ras antibodies (#67648), Ras-GRF antibodies (#3322), p65-NF-κB antibodies (#8242), and phospho- (p-)p65-NF-κB antibodies (#3033). Anti-mouse CDK4 (#sc-56,277) and CDK6 antibodies (#sc-56,277), were purchased from Santa Cruz Biotechnology (Santa Cruz, CA, USA). These primary antibodies were diluted 1:800 to 1:1000 in Tris-buffered saline containing 0.1% of Tween 20 and incubated with the PVDF membranes at 37 °C for 60 min. To detect the primary antibody, the PVDF membranes were probed for 1 h with a horseradish peroxidase-conjugated anti-rabbit or anti-goat IgG antibody diluted 1:5000 to 1:10000 in TBST. Immunoreactive protein bands were visualized with the Enhanced Chemiluminescence (ECL) Reagent Kit (Millipore, USA) and documented using a Western-Light Chemiluminescent Detection System (Applied Biosystems, USA). Densitometric analyses of the protein bands were performed in the ImageJ software (version 1.48, National Institutes of Health, USA). All fold changes of band densities were determined with normalization to GAPDH, an endogenous control. Relative protein expression was calculated as relative density of a protein band normalized to the endogenous control. Each experiment was conducted in triplicate and repeated three times independently.

### Real-time PCR analysis

Total RNA was extracted from the pretreated cells using the RNA Extraction Kit (Toyobo, Japan). Then, the total RNA was reverse-transcribed into cDNA by means of the cDNA Reverse Transcription Kit (Invitrogen, USA). The UPL probe No. of Orai1 is #108 (cat. No. 04692276001), the UPL probe No. of cyclin D1 is #72 (cat. No. 04688953001), the UPL probe No. of cyclin E is #108 (cat. No. 04692276001), the UPL probe No. of CDK4 is #50 (cat. No. 04688112001), and the UPL probe No. of CDK6 is #15 (cat. No. 04685148001). The forward primer for Orai1 expression analysis was 5`-TACTTAAGCCGCGCCAAG-3`, and the reverse primer was 5`-ACTTCCACCATCGCTACCA-3`. The forward primer for cyclin D1 expression analysis was 5`-TTTCTTTCCAGAGTCATCAAGTGT-3`, and the reverse primer was 5`- TGACTCCAGAAGGGCTTCAA-3`. The forward primer for cyclin E expression analysis was 5`-TTTCTGCAGCGTCATCCTC-3`, and the reverse primer was 5`-TGGAGCTTATAGCTTCGCACA-3`. The forward primer for CDK4 expression analysis was 5`- TCAGTGGTGCCAGAGATGG-3`, and the reverse primer was 5`-GGAAGGCAGAGATTCGCTTA-3`. The forward primer for CDK6 expression analysis was 5`- GCCCTTACCTCGGTGGTC-3` and the reverse primer was 5`-ACAGGGGTGGCATAGCTG-3`. Real-time PCR was used to measure mRNA expression levels of Orai1, cyclin D1, cyclin E, CDK4, and CDK6. Real-time PCR was carried out with the SYBR green PCR reagent (Takara, Japan) on a Stratagene M63005P Multiplex Quantitative PCR System (Agilent Technologies, Germany). GAPDH served as an endogenous control. The real-time PCR cycling conditions were as follows: denaturation at 95 °C for 10 min; 35 cycles of denaturation at 95 °C for 10 s, annealing at 59 °C for 15 s, and extension at 72 °C for 20 s; and final extension at 72 °C for 15 min. The reaction was terminated by cooling at 4 °C. Each experiment was conducted in triplicate and repeated three times independently.

### Cell proliferation assay

Cell proliferation was analyzed by a 3-(4,5-dimethylthiazol-2-yl)-2,5- diphenyltetrazolium bromide (MTT) assay. Approximately 10^4^ cells per well were added into a 96-well cell culture plate. After the cells were cultured for 24, 48, 64, or 72 h, 50 μL of MTT (Sigma-Aldrich, USA) and 150 μL of the culture medium were added into each well of the cell culture plate. Then, the cell culture plates were incubated at 37 °C for 4 h. MTT was dissolved in 200 μL of DMSO in each well for 30 min. Finally, the absorbance of each well was measured at 570 nm on a microplate reader (BioAssay Systems, USA). All fold changes of cell proliferation were normalized according to the following formula: (Sample absorbance - Blank absorbance)/(Control absorbance - Blank absorbance). Each experiment was carried out in triplicate and repeated three times independently.

### Cell apoptosis assay

Apoptosis of osteoblasts was detected using a DeadEnd Fluorometric Tunel System (Promega, USA) that end-labels fragmented DNA from apoptotic cells via modified terminal deoxynucleotidyl transferase dUTP nick end labeling (Tunel) staining. Briefly, the cells were fixed with a 4% methanol-free formaldehyde solution and rinsed with PBS twice, then the fragmented DNA of the apoptotic cells was end-labeled with fluorescein-12-dUTP, and the slides were incubated with 4,6-diamidino-2-phenylindole (DAPI) (Pierce, Illinois, USA) to stain nuclei. The cells were immediately analyzed under a fluorescence microscope with a standard fluorescein filter set (Olympus, Japan) to examine green fluorescence of apoptotic cells at 520 nm and blue fluorescence of DAPI-stained nuclei at 460 nm. Each experiment was carried out in triplicate and repeated three times independently.

### Cell cycle assay

The cells were cultured in the serum-free medium for 24 h. Then, the cells were cultivated in the medium with 10% of FBS. After 24 h, the cells were collected into a centrifuge tube and resuspended in 1 mL of anhydrous ethanol. The cells were incubated overnight at 4 °C. Next, the cells were collected into a centrifuge tube and resuspended in 500 μL of a propidium iodide (PI) solution (Absin, China). The cells were stained in at 4 °C (in a refrigerator) for 60 min. Finally, the cell cycle was examined by flow cytometry, and the data were analyzed statistically. Each experiment was conducted in triplicate and repeated three times independently.

### Application of 2-APB

The 2-Aminoethyl Diphenylborinate (2-APB) stock solution (Sigma-aldrich, USA) was diluted to a concentration of 50 μM in the cell culture medium (Gibco, USA). DMSO (Sigma-aldrich, USA) alone was dissolved as negative (vehicle) control. After that, cell proliferation and cell cycle assays were performed at an appropriate period.

### Application of ARS-853

The ARS-853 stock solution (Glpbio, USA) was diluted to a concentration of 25 μM in the cell culture medium (Gibco, USA). DMSO (Sigma-aldrich, USA) alone was dissolved as negative (vehicle) control. After that, western blotting, cell proliferation and cell cycle assays were performed after incubation with or without the Ras signaling pathway inhibitor ARS-853 (25 nM) for an appropriate period.

### Statistical analysis

We employed SPSS 16.0 software to analyze the data statistically. All values are expressed as mean ± standard error from at least three independent experiments. Multigroup comparisons were analysed using one-way (ANOVA) followed by Tukey’s post hoc significance difference tests. Student’s t test was used for pairwise comparisons. The results were considered statistically significant when a *p* value was < 0.05.

## Results

### Expression of Orai1 is inhibited by Orai1 siRNA in MC3T3-E1 cells

**Fig. 1:**
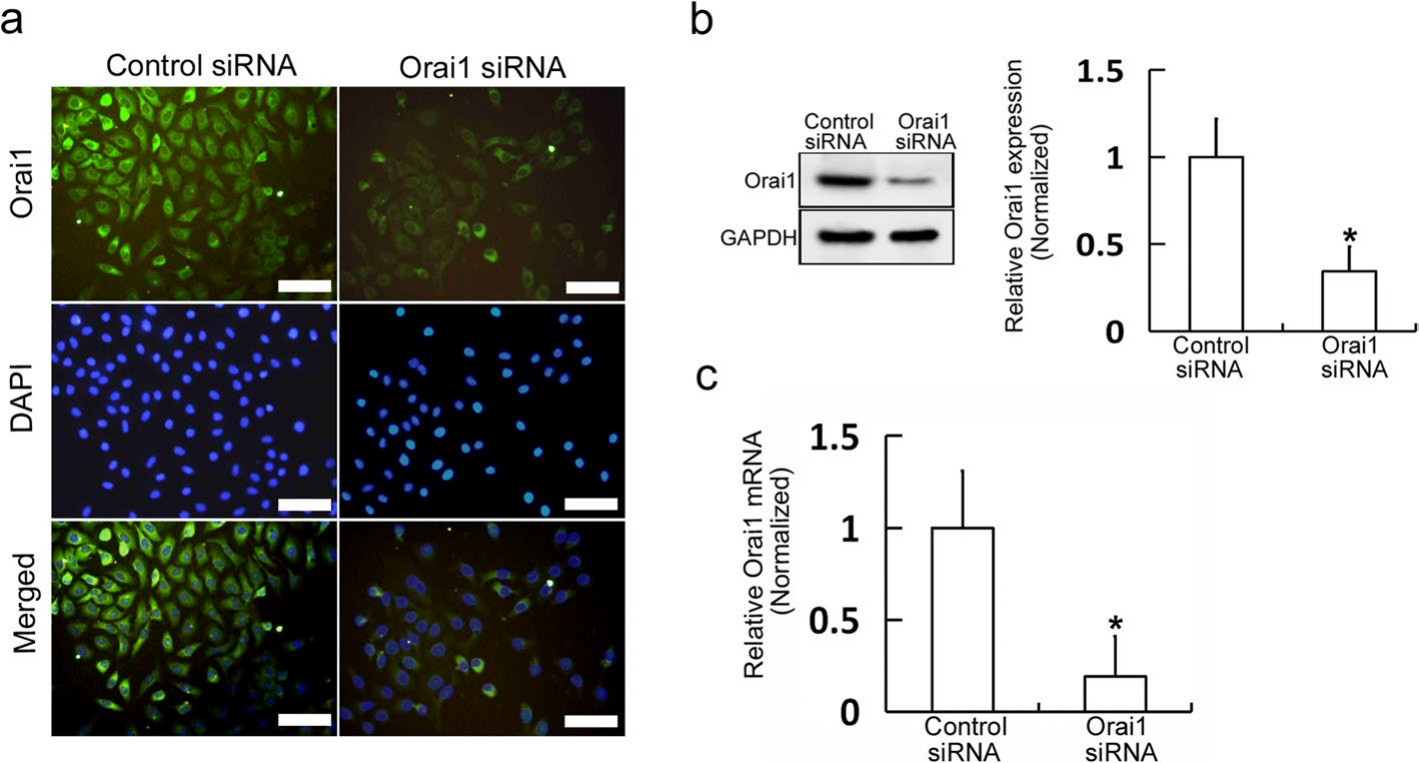
The expression of Orai1 is inhibited by Orai1 siRNA in MC3T3-E1 cells. **a** Orai1 protein levels were examined by immunofluorescence analysis in MC3T3-E1 cells transfected with either control siRNA or Orai1 siRNA (Scale =100 μm). **b** Orai1 protein levels were examined by western blot analysis in MC3T3-E1 cells transfected with either control siRNA or Orai1 siRNA. **c** The Orai1 mRNA levels were examined by real-time PCR in MC3T3-E1 cells transfected with either control siRNA or Orai1 siRNA. Data are presented as means ± SD from three independent experiments. *p < 0.05 as compared with the control

Some studies indicate that the deletion of the Orai1 gene can lead to a phenotype of osteoporosis, e.g., decreased bone density in mice, suggesting that Orai1 is involved in the pathogenesis of osteoporosis [6, 7]. To investigate the role of Orai1 in the onset and development of osteoporosis via the regulation of the proliferation of osteoblasts, Orai1 siRNA was transfected into MC3T3-E1 cells to reduce the expression of Orai1. We transfected a control siRNA as negative control. Immunofluorescence and western blotting were performed to assess Orai1 protein expression, and real-time PCR was carried out to evaluate Orai1 mRNA expression after the Orai1 siRNA transfection. We found that Orai1 protein expression levels were remarkably decreased by the Orai1 siRNA transfection (*p* = 0.003, F = 0.174, df = 3, Fig. 1a, b and Additional file 1). Moreover, Orai1 mRNA levels were remarkably decreased by the Orai1 siRNA transfection (*p* = 0.000, F = 0.294, df = 3, Fig. 1c and Additional file 1). The results indicated that Orai1 siRNA successfully inhibited Orai1 expression in MC3T3-E1 cells.

### Orai1 downregulation reduces proliferation of MC3T3-E1 cells and causes cell cycle arrest

**Fig. 2:**
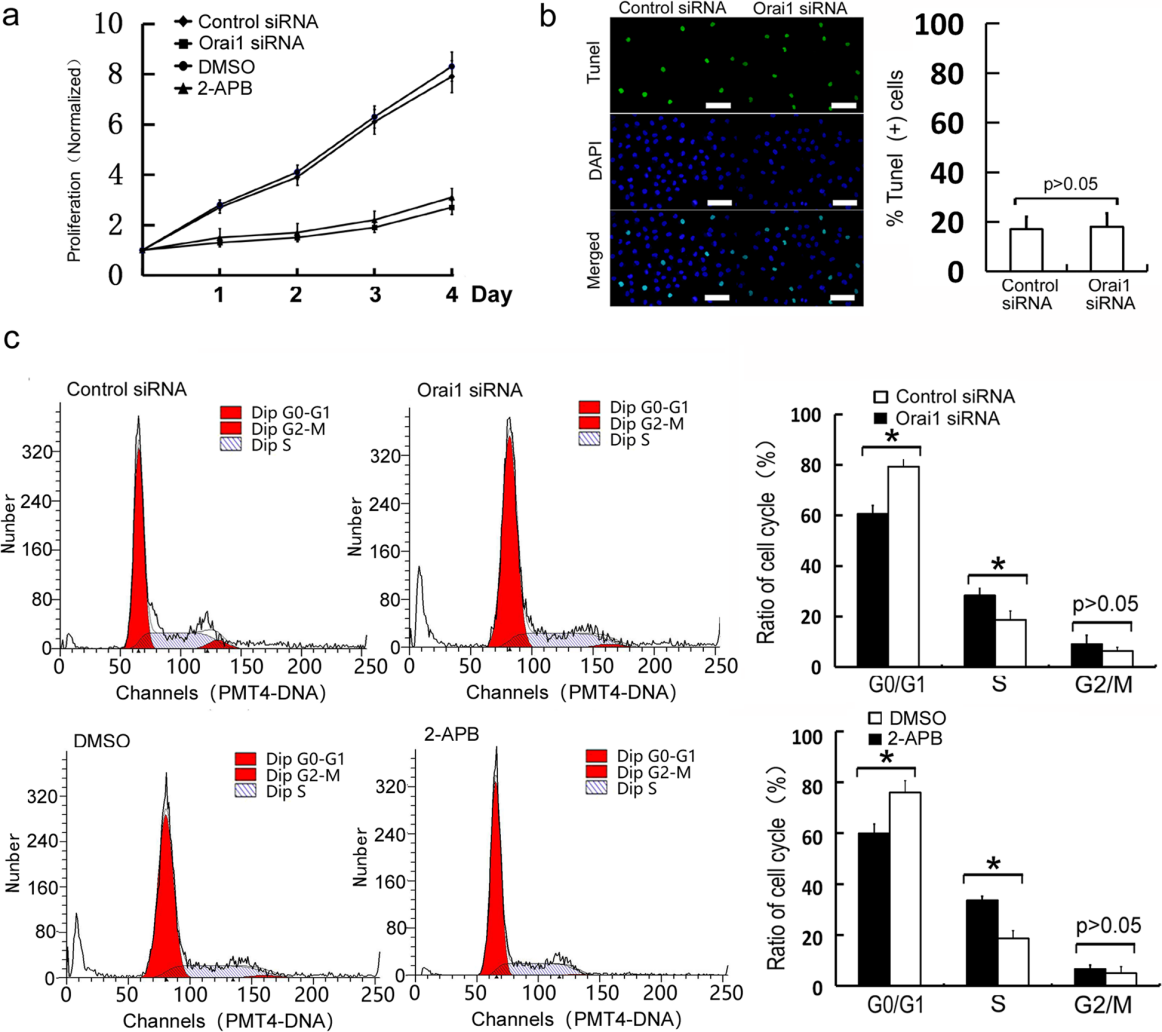
Orai1 downregulation reduces the proliferation of MC3T3-E1 cells and causes their cell cycle arrest. Control siRNA and Orai1 siRNA transfected into MC3T3-E1 cells for an appropriate period. The 2-APB stock solution was diluted to a concentration of 50 μM in the cell culture medium. DMSO alone was dissolved as negative (vehicle) control. After that, cell proliferation and cell cycle assays were performed at an appropriate period. **a** The cell proliferation was evaluated by the MTT assay of MC3T3-E1 cells transfected with either control siRNA, Orai1 siRNA, DMSO or 2-APB. **b** Apoptosis was evaluated by Tunel staining of the MC3T3-E1 cells transfected with either control siRNA or Orai1 siRNA (Scale =100 μm). **c** The cell cycle distribution was investigated using PI staining of the MC3T3-E1 cells transfected with either control siRNA, Orai1 siRNA, DMSO or 2-APB. (Data are presented as means ± SD from three independent experiments. *p < 0.05 as compared with the control

A decrease in the number of osteoblasts occurs during the development of osteoporosis. Therefore, the proliferative ability of osteoblasts plays an important role in the progression of osteoporosis. To test whether Orai1 participates in the regulation of the proliferation of osteoblasts, Orai1 siRNA was utilized to suppress the expression of Orai1 in MC3T3-E1 cells. Then, changes in the proliferation of MC3T3-E1 cells were detected by the MTT assay. We noticed that the proliferation of MC3T3-E1 cells significantly decreased in a time-dependent manner after the Orai1 downregulation (*p* = 0.002, F = 0.804, df = 3, Fig. 2a). Apoptosis is another important cause of the reduction in the osteoblast number. Alterations of the apoptosis of MC3T3-E1 cells were assessed by Tunel staining. There were no significant changes in the apoptosis of osteoblasts after the Orai1 downregulation (*p* = 0.407, F = 0.906, df = 3, Fig. 2b). This result confirmed that the Orai1 knockdown reduced the proliferation of osteoblasts. The process of cell proliferation depends on the switching of phases of the cell cycle. The S phase is the DNA synthesis phase, which is an important indicator of the cellular proliferative ability. To determine whether Orai1 is involved in the regulation of osteoblast proliferation, we applied PI staining and flow cytometry to detect cell cycle changes in the osteoblasts after the Orai1 knockdown. The results meant that the percentages of cells at the G0-G1 phase significantly increased after the Orai1 downregulation (*p* = 0.003, F = 0.878, df = 3, Fig. 2c). And the percentages of cells at the S phase significantly decreased after the Orai1 downregulation (*p* = 0.004, F = 0.114, df = 3, Fig. 2c). To further confirm those effects caused by silencing Orai1 were were consistent with blocking store operated Ca^2+^ entry, 50 μM 2-APB were used, which were found blocking store operated Ca^2+^ entry in several different cell lines. Then, the proliferation and cell cycle of MC3T3-E1 cells were detected. We found that the proliferation evidently decreased in MC3T3-E1 cells which were treated with 50 μM 2-APB (*p* = 0.000, F = 0.742, df = 3, Fig. 2a). And the S phase of cell cycle evidently decreased in MC3T3-E1 cells which were treated with 50 μM 2-APB (*p* = 0.002, F = 0.678, df = 3, Fig. 2c). The results confirmed that those effects caused by silencing Orai1 were consistent with blocking store operated Ca^2+^ entry. The above results suggested that the Orai1 downregulation reduces the entry into the S phase and inhibits the proliferation of osteoblasts, resulting in a decreased number of osteoblasts.

### The Orai1 knockdown reduces the expression of cyclin D1 in MC3T3-E1 cells

**Fig. 3:**
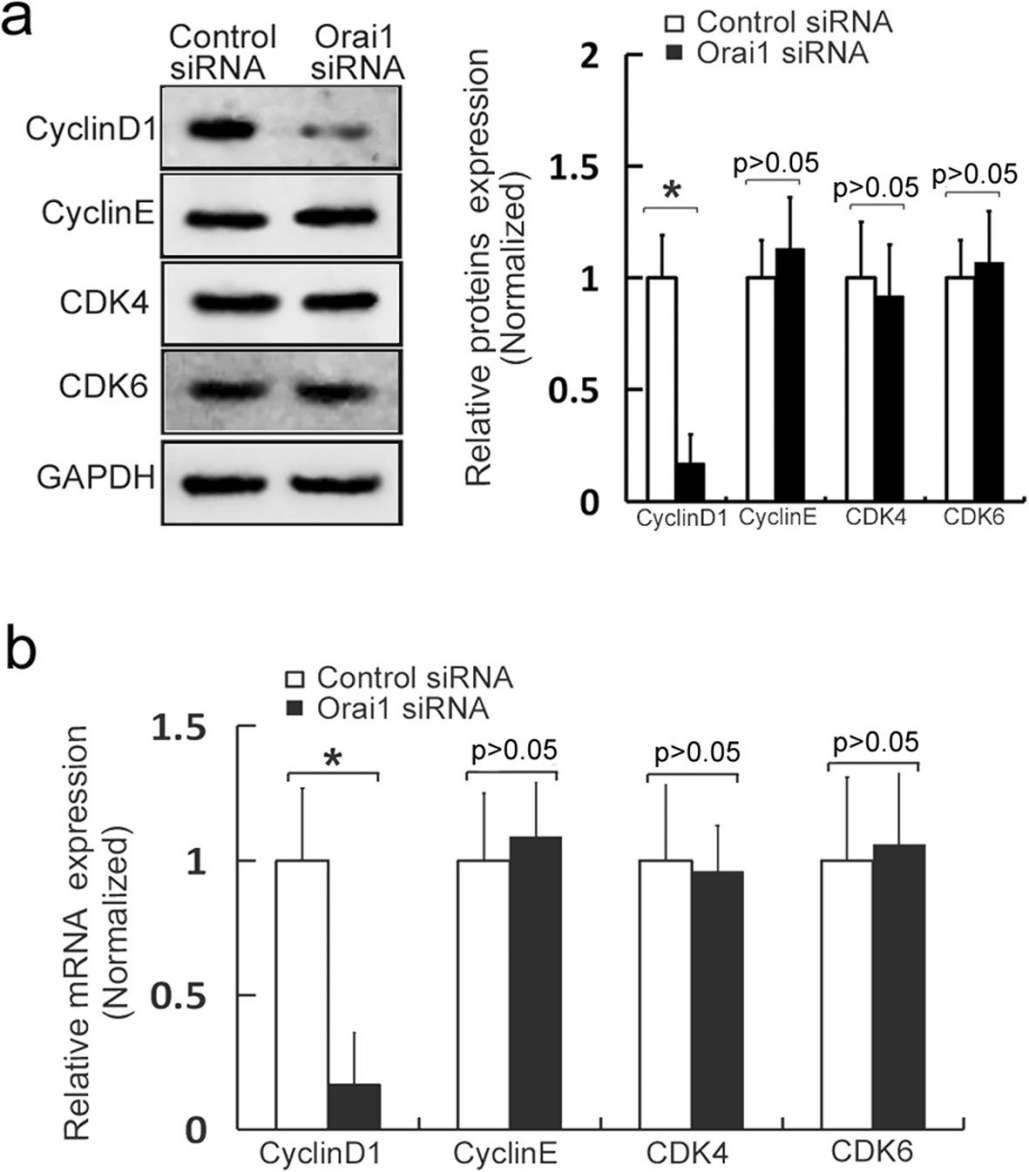
Orai1 downregulation lowers the expression of cyclin D1 in MC3T3-E1 cells. **a** Western blotting was performed to assess the expression levels of cyclin D1, cyclin E, CDK4, and CDK6 in the MC3T3-E1 cells transfected with either control siRNA or Orai1 siRNA. **b** Real-time PCR was carried out to measure mRNA levels of cyclin D1, cyclin E, CDK4, and CDK6 in the MC3T3-E1 cells transfected with either control siRNA or Orai1 siRNA. Data are presented as means ± SD from three independent experiments. *p < 0.05 as compared with the control

The progression of the cell cycle depends on two key types of regulatory molecules, cyclins and cyclin-dependent kinases (CDKs) [8, 9]. To investigate whether Orai1 plays a part in the regulation of the cell cycle in osteoblasts, the expression of cyclin D1, cyclin E, CDK4, and CDK6 was measured by western blotting after the Orai1 downregulation. We found that the expression of cyclin D1 in MC3T3-E1 cells significantly decreased after the Orai1 knockdown (*p* = 0.001, F = 0.097, df = 3, Fig. 3a and Additional file 2). There was no significant change in the expression of cyclin E, CDK4, and CDK6 in osteoblasts after the Orai1 downregulation (*p* = 0.203, F = 0.376, df = 3, *p* = 0.454, F = 0.154, df = 3 and *p* = 0.374, F = 0.284, df = 3, respectively, Fig. 3a and Additional file 2). Then, the mRNA levels of cyclin D1, cyclin E, CDK4, and CDK6 were evaluated by real-time PCR after the Orai1 downregulation. These results indicated that the mRNA level of cyclin D1 was remarkably reduced by the Orai1 downregulation (*p* = 0.002, F = 0.682, df = 3, Fig. 3b). There were no significant changes in the mRNA levels of cyclin E, CDK4, and CDK6 in osteoblasts after the Orai1 downregulation (*p* = 0.169, F = 0.244, df = 3, *p* = 0.603, F = 0.721, df = 3 and *p* = 0.186, F = 0.958, df = 3, respectively, Fig. 3b). These results confirmed that the Orai1 downregulation can significantly reduce the expression of cyclin D1, thereby leading to osteoblast cell cycle arrest and a proliferation reduction.

### Orai1 downregulation reduces the proliferation of osteoblasts by inactivating the Ras-NF-κB signaling pathway

**Fig. 4:**
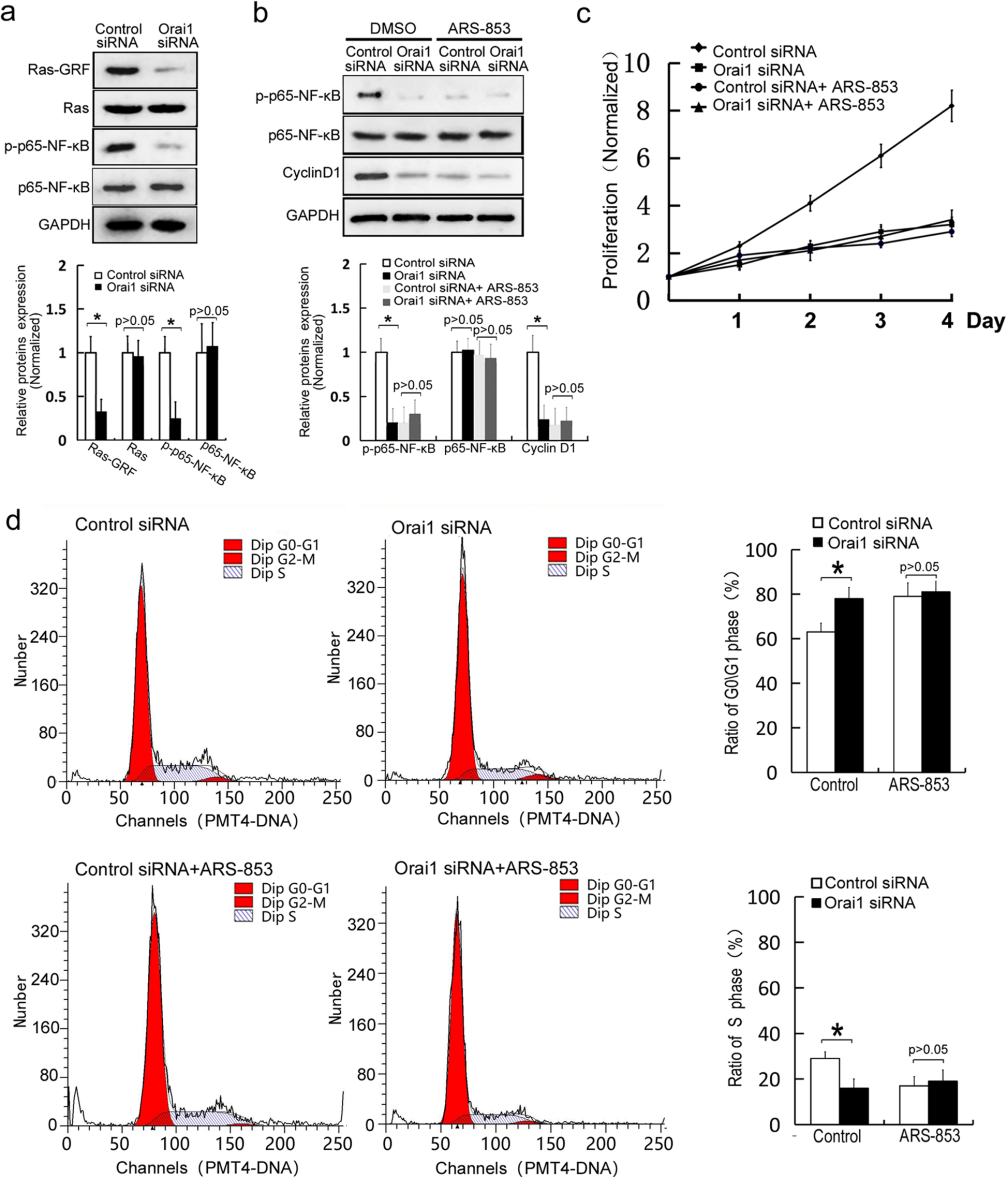
Orai1 downregulation reduces the proliferation of osteoblasts by inactivating the Ras-NF-κB signaling pathway. **a** The amounts of Ras-GRF and p-p65-NF-κB were measured in the MC3T3-E1 cells transfected with either control siRNA or Orai1 siRNA. ARS-853 was dissolved in DMSO to prepare a stock solution. After MC3T3-E1 cells were transfected with either the control siRNA or Orai1 siRNA, the ARS-853 stock solution was diluted to a concentration of 25 nM in the cell culture medium. DMSO alone was dissolved as negative (vehicle) control. After that, western blotting and cell proliferation and cell cycle assays were performed after incubation with or without the Ras signaling pathway inhibitor ARS-853 (25 nM) for an appropriate period. **b** After incubation with or without ARS-853, the amounts of p-p65-NF-κB and cyclin D1 were evaluated in the MC3T3-E1 cells transfected with either the control siRNA or Orai1 siRNA. **c** After incubation with or without ARS-853, the proliferation rate of the MC3T3-E1 cells (transfected with either the control siRNA or Orai1 siRNA) was evaluated. **d** After incubation with or without ARS-853, the cell cycle distribution was investigated for the MC3T3-E1 cells transfected with either the control siRNA or Orai1 siRNA. Data are presented as means ± SD from three independent experiments. *p < 0.05 as compared with the control

It has been reported that inhibition of the Ras-NF-κB signaling pathway leads to a reduction in osteoblast proliferation [10–12]. Some studies have revealed that calcium ions activate the Ras signaling cascade by directly binding to Ras-GRF [13]. To further clarify the mechanism of Orai1 action in the regulation of osteoblast proliferation, alterations in the amounts of Ras-GRF and p-p65-NF-κB, which are activated forms of Ras and NF-κB, were studied after the Orai1 downregulation. We did not detect any significant change in the amounts of total Ras and p65-NF-κB after the Orai1 downregulation. Nonetheless, we found that the presence of Ras-GRF and p-p65-NF-κB were significantly reduced by the Orai1 downregulation (*p* = 0.011, F = 0.256, df = 3 and *p* = 0.003, F = 0.128, df = 3, respectively, Fig. 4a and Additional file 3). The results suggested that Orai1 downregulation can reduce the activation of Ras and NF-κB in MC3T3-E1 cells. To further elucidate whether the Orai1 downregulation can inactivate the Ras-NF-κB signaling pathway to reduce osteoblast proliferation, the Ras signaling pathway inhibitor ARS-853 was applied. The ARS-853 stock solution was diluted to a concentration of 25 nM in the cell culture medium. DMSO alone was dissolved as negative (vehicle) control. After that, western blotting and cell proliferation and cell cycle assays were performed after incubation with or without the Ras signaling pathway inhibitor ARS-853 (25 nM) for an appropriate period. The expression of p-p65-NF-κB and cyclin D1 was decreased by the Orai1 downregulation (*p* = 0.003, F = 0.112, df = 3 and *p* = 0.009, F = 0.209, df = 3, respectively, Fig. 4b and Additional file 4). On the other hand, the addition of ARS-853 reduced the amounts of p-p65-NF-κB and cyclin D1 in the control siRNA transfection groups to the levels comparable to the level in the Orai1 siRNA transfection groups. The inhibition of Ras signaling pathway did not further reduce the the amounts of p-p65-NF-κB and cyclin D1 in Orai1 siRNA groups.(*p* = 0.339, F = 0.758, df = 3 and *p* = 0.249, F = 0.778, df = 3, respectively, Fig. 4b). Furthermore, we noted that Orai1 siRNA significantly reduced cell proliferation in the control group (*p* = 0.002, F = 0.570, df = 3, Fig. 4c). Nevertheless, the cell proliferation in control siRNA groups was similar to that in Orai1 siRNA groups after ARS-853 was added (*p* = 0.443, F = 0.506, df = 3, Fig. 4c). The that inhibition of Ras signaling pathway did not further reduce the proliferation in Orai1 siRNA groups. Moreover, we demonstrated that Orai1 siRNA significantly diminished the proportion of cells in the S phase in the control group (*p* = 0.026, F = 0.183, df = 3, Fig. 4d). Nonetheless, we found that the proportion of cells in the S phase in control siRNA groups was similar to that in Orai1 siRNA groups after ARS-853 was added. The inhibition of Ras signaling pathway did not further reduce the proportion of cells in the S phase in Orai1 siRNA groups. (*p* = 0.883, F = 0.813, df = 3, Fig. 4d). The above results indicated that the activity of the Ras-NF-κB signaling pathway is required for Orai1-regulated proliferation of MC3T3-E1 cells. These results confirmed that the Orai1 downregulation reduced the proliferation of osteoblasts by inactivating the Ras-NF-κB signaling pathway.

## Discussion

Osteoporosis is a common degenerative disease of the skeletal system and seriously affects the health of the elderly. The inhibition of osteoblast proliferation is an important reason for the onset and development of osteoporosis [1–5]. Therefore, further research on the mechanism regulating osteoblast proliferation should reveal a new biological treatment of clinical osteoporosis.

Orai1 is mainly composed of four transeptal segments and is a quaternary transmembrane protein. It is located on the plasma membrane, and its C and N termini are both located in the cell. The first segment of Orai1 is rich in proline and arginine, and the third transeptal segment is rich in glutamate, which can selectively bind and transport calcium ions. When extracellular stimuli deplete endoplasmic-reticulum calcium, STIM1 located on the endoplasmic omentum can approach Orai1 and induce it to form a calcium channel that promotes calcium ion influx and activates calcium signaling cascades to complete some cellular physiological functions [8, 9]. Previous studies have revealed that Orai1 may be a target for the treatment or prevention of the bone loss caused by osteoclasts in the RAW cell line. Orai1 knockdown reduces SOCE and inhibits RANKL-induced osteoclastogenesis of RAW264.7 cells, a murine monocyte/macrophage cell line, by suppressing the induction of NFATc1 [10]. Osteoblast differentiation and function are affected by Orai1 deficiency. Deletion of the Orai1 gene can lead to a phenotype of osteoporosis, e.g., decreased bone density in mice, suggesting that Orai1 participates in the pathogenesis of osteoporosis [11–13]. On the other hand, the effects of Orai1 on osteoblasts and its cumulative influence on the stability of the intraosseous environment have not been fully studied; this situation limits our understanding of Orai1 functions in bone biology. Inhibition of osteoblast proliferation leads to a decrease in the number of osteoblasts, which is an important reason for the onset and development of osteoporosis. To investigate whether Orai1 is involved in the proliferation of osteoblasts, we transfected Orai1 siRNA into MC3T3-E1 cells. We found that the proliferation of osteoblasts was significantly reduced by the Orai1 downregulation in MC3T3-E1 cells. Moreover, we noted that there were no significant changes in apoptosis of MC3T3-E1 cells after the Orai1 downregulation. These results confirm that the Orai1 downregulation reduces the proliferation of osteoblasts. The process of cell proliferation depends on the switching of cell cycle phases. The S phase of the cell cycle is the DNA synthesis phase, which is an important indicator of the cellular proliferative ability. In this study, we examined changes in the cell cycle of osteoblasts after the Orai1 downregulation. The results revealed that the proportion of cells at the G0-G1 transition of the cell cycle significantly increased and the proportion of cells in the S phase significantly decreased after the downregulation of Orai1. These results mean that an Orai1 knockdown can reduce the proliferation of osteoblasts, thereby decreasing their number.

The progression of the cell cycle depends on two key types of regulatory molecules, cyclins and CDKs. The latter are multifunctional enzymes that phosphorylate various protein substrates during cell cycle progression, especially when the cell cycle advances from the G1 phase to S phase. Cyclins, including cyclins D1 and E, can bind to and activate CDK4 and CDK6, playing a key role in the progression of the cell cycle from the G1 phase to S phase [14, 15]. CDK activity is also negatively regulated by cyclin-dependent kinase inhibitors. P27 is a key member of the cyclin-dependent kinase inhibitor family and binds to the cyclin-CDK2 complex in the nucleus and negatively regulates the cell cycle, thus leading to cell cycle arrest in the G1 phase [16, 17]. We found that protein expression and mRNA levels of cyclin E, CDK4, and CDK6 in osteoblasts are not significantly changed by the Orai1 downregulation, whereas the protein amount and mRNA level of cyclin D1 in osteoblasts are significantly reduced by the Orai1 downregulation. These results suggest that the Orai1 knockdown can reduce the expression of cyclin D1 in osteoblasts and arrest the cell cycle between the G1 phase and S phase, thereby slowing the proliferation of osteoblasts.

The transcriptional activation of cyclin D1 is regulated by many homeopathic elements such as CRE, AP-1 and SP-1. It has also been reported that the expression of cyclin D1 is not only positively correlated with the expression of NF- κB, but also NF- κB is involved in the transcriptional regulation of cyclin D1. Karim et al. found that the promoter region of cyclin D1 contained NF- κB binding site [18–20]. It has been demonstrated that the suppression of the Ras-NF-κB signaling pathway slows osteoblast proliferation. Growth factors and other mitotic stimuli can induce the expression of cyclin D1 through the Ras-NF-κB signaling pathway [21–23]. Ras is a small GTPase that cycles between a GTP-bound active conformation and a GDP-bound inactive conformation. Ras-GRF is a Ras effector proteins, which aid in the integration of Ca^2+^ and Ras. It was shown that the Ras-GRF contains calmodulin (CaM) binding motifs and activates Ras in a Ca^2+^-dependent manner [24, 25]. The transcription factor NF-κB is a critical regulator of osteoblast proliferation. The most common form of NF-κB is the heterodimer of p50-NF-κBand p65-NF-κB. In resting state, the NF-κB dimers remain in the cytoplasm as an inactive complex with the IκΒ inhibitory proteins. When IκB proteins are ubiquitinated and degradated by proteasomes, p65-NF-κB is phosphorylated and released from the inhibitory complex. The phosphorylated p65-NF-κB then binds specific nucleotide sequence of the target genes and mediates target genes transcription [26]. The expression of Ras-GRF and p-p65-NF-κB represents the activity of Ras-NF-κB signaling pathway in osteoblasts. Protein expression is a multistage process that involves the mRNA transcription, translation and turnover of mRNA and proteins, so mRNA transcription levels are not sufficient to predict protein levels in many scenarios [27]. In order to accurately detect protein expression levels, we measured the expression of Ras-GRF, Ras, p65-NF-κB, and p-p65-NF-κB by Western blot rather than PCR in the study. We found that the presence of Ras-GRF and p-p65-NF-κB were significantly reduced by the Orai1 downregulation. These data suggest that the Orai1 downregulation can reduce the expression of cyclin D1 in osteoblasts by inactivating the Ras-NF-κB signaling pathway. To verify this result, the Ras signaling pathway inhibitor ARS-853 was used. The findings revealed that inhibition of Ras signaling pathway did not further reduce the proliferation, the proportion of cells in the S phase, and the expression of cyclin D1 in Orai1 siRNA groups. These results confirm that the Orai1 downregulation reduces the proliferation of osteoblasts by inactivating the Ras-NF-κB signaling pathway.

## Limitations

All the results of this study were from a single osteoblast cell line, and it is necessary to validate these findings in more osteoblast cell lines. Due to the limitations of in vitro studies, this study cannot simulate the molecular interactions in complex environment in vivo. This may make it difficult for this study to accurately predict the potential interactions between molecules.

## Conclusions

Our study indicates that Orai1 downregulation leads to a slowdown of osteoblast proliferation and to cell cycle arrest. The Orai1 downregulation inactivates the Ras-NF-κB signaling pathway to reduce the expression of cyclin D1 thus decreasing the proliferation of osteoblasts. Abnormal Orai1 expression or function may be a major cause of osteoporosis development. Therefore, detailed research on the role of Orai1 in the onset of osteoporosis can yield a new idea for the biological treatment of osteoporosis.

## Supplementary Information

**Additional file 1. **The raw data of western blot of Orai1 and GAPDH. **a** Orai1 protein levels and (**b**) GAPDH protein levels were examined by western blot analysis in MC3T3-E1 cells transfected with either control siRNA or Orai1 siRNA. GAPDH was used as an endogenous control.

**Additional file 2. **The raw data of western blot of cyclin D1, cyclin E, CDK4, CDK6 and GAPDH. **a** cyclin D1 protein levels, (**b**) cyclin E protein levels, (**c**) CDK4 protein levels, (**d**) CDK6 protein levels and (**e**) GAPDH protein levels were examined by western blot analysis in MC3T3-E1 cells transfected with either control siRNA or Orai1 siRNA. GAPDH was used as an endogenous control.

**Additional file 3. **The raw data of western blot of Ras-GRF, Ras, p-p65-NF-κB, p65-NF-κB and GAPDH. **a** Ras-GRF protein levels, (**b**) Ras protein levels, (**c**) p-p65-NF-κB protein levels, (**d**) p65-NF-κB protein levels (**e**) GAPDH protein levels were examined by western blot analysis in MC3T3-E1 cells transfected with either control siRNA or Orai1 siRNA. GAPDH was used as an endogenous control.

**Additional file 4. **The raw data of western blot of p-p65-NF-κB, p65-NF-κB, Cyclin D1 and GAPDH. After incubation with or without ARS-853, the amounts of (**a**) p-p65-NF-κB protein levels, (**b**) p65-NF-κB protein levels, (**c**) cyclin D1 protein levels and (**d**) GAPDH protein levels were evaluated in the MC3T3-E1 cells transfected with either the control siRNA or Orai1 siRNA. GAPDH was used as an endogenous control.

## Data Availability

The datasets used and/or analysed during the current study available from the corresponding author on reasonable request.

## Abbreviations

CRAC: Calcium release-activated calcium channel
STIM1: Stromal interaction molecule 1
SOCE: Store-operated calcium entry
ATCC: American Type Culture Collection
siRNA: Small interfering RNA
DAPI: 4,6-diamidino-2-phenylindole
PVDF: Polyvinylidene fluoride microporous
ECL: Enhanced Chemiluminescence
MTT: 3-(4,5-dimethylthiazol-2-yl)-2,5- diphenyltetrazolium bromide
PI: Propidium iodide
2-APB: 2-Aminoethyl Diphenylborinate
CDKs: Cyclin-dependent kinases
NF-κB: Nuclear factor kappa-B
