# Supplementary material for: Orai1 downregulation causes proliferation reduction and cell cycle arrest via inactivation of the Ras-NF-κB signaling pathway in osteoblasts
Source: BMC Musculoskelet Disord. 2022 Apr 11;23:347. doi: 10.1186/s12891-022-05311-y (PMC8996479; doi:10.1186/s12891-022-05311-y)

Uncropped Western blot images

Additional file 1 The raw data of western blot of Orai1 and GAPDH. (a) Orai1 protein levels and (b) GAPDH protein levels were examined by western blot analysis in MC3T3-E1 cells transfected with either control siRNA or Orai1 siRNA. GAPDH was used as an endogenous control.

a

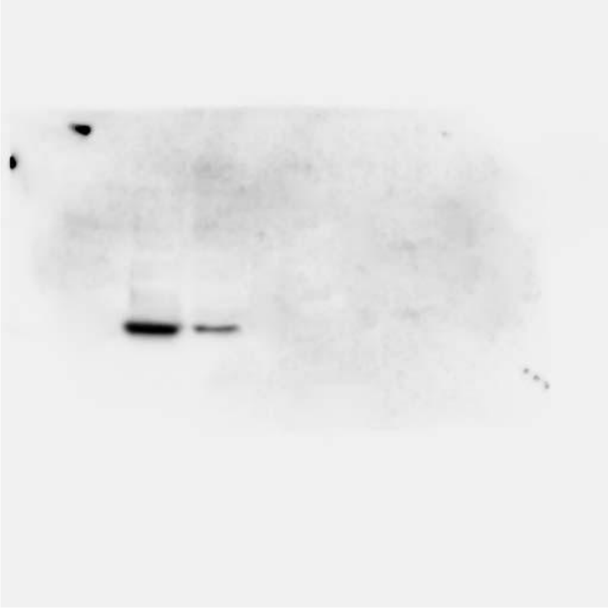

b

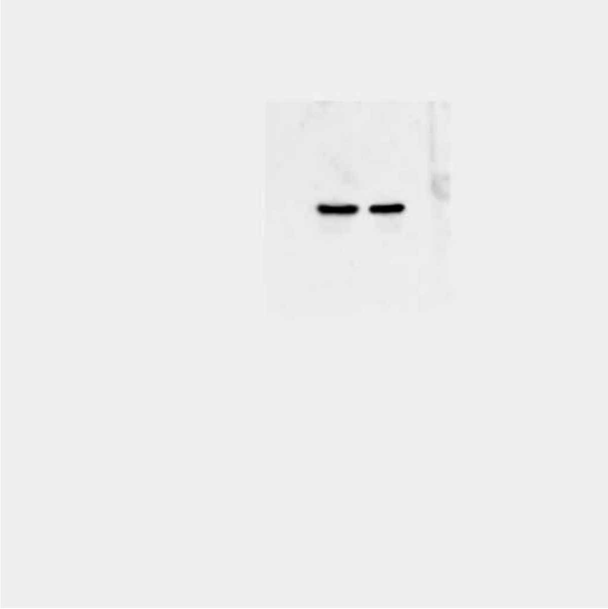

Supplement: Supplementary file 1 — Additional file 1. The raw data of western blot of Orai1 and GAPDH. a Orai1 protein levels and (b) GAPDH protein levels were examined by western blot analysis in MC3T3-E1 cells transfected with either control siRNA or Orai1 siRNA. GAPDH was used as an endogenous control. [file 12891_2022_5311_MOESM1_ESM.pdf]
