# Supplementary material for: Orai1 downregulation causes proliferation reduction and cell cycle arrest via inactivation of the Ras-NF-κB signaling pathway in osteoblasts
Source: BMC Musculoskelet Disord. 2022 Apr 11;23:347. doi: 10.1186/s12891-022-05311-y (PMC8996479; doi:10.1186/s12891-022-05311-y)

Uncropped Western blot images

Additional file 2 The raw data of western blot of cyclin D1, cyclin E, CDK4, CDK6 and GAPDH. (a) cyclin D1 protein levels, (b) cyclin E protein levels, (c) CDK4 protein levels, (d) CDK6 protein levels and (e) GAPDH protein levels were examined by western blot analysis in MC3T3-E1 cells transfected with either control siRNA or Orai1 siRNA. GAPDH was used as an endogenous control.

a

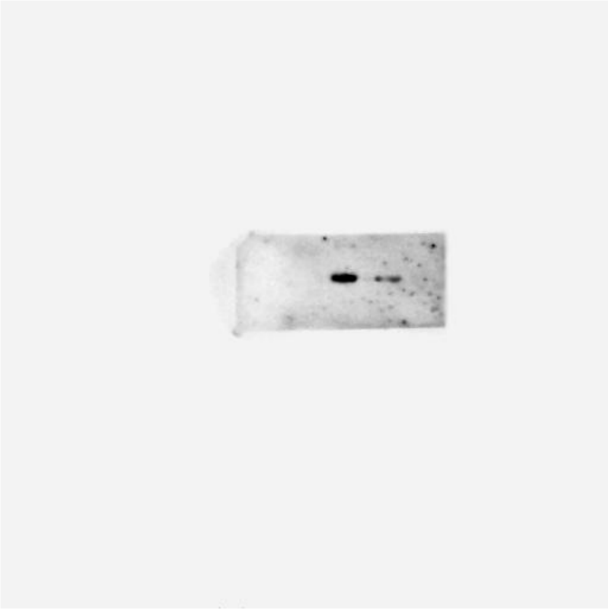

b

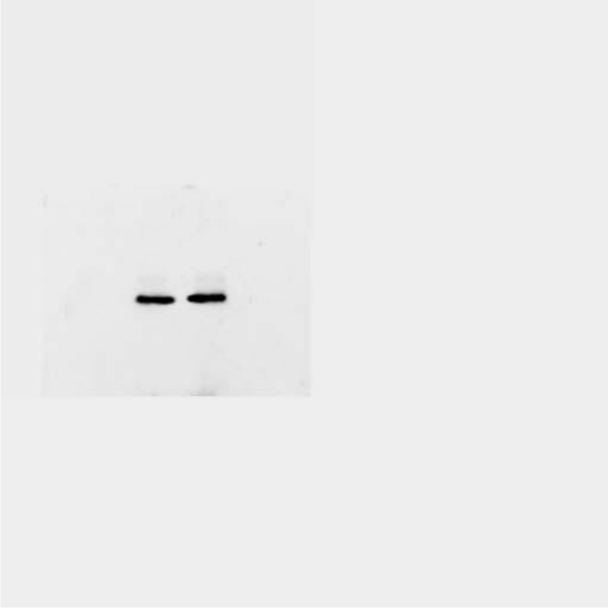

c

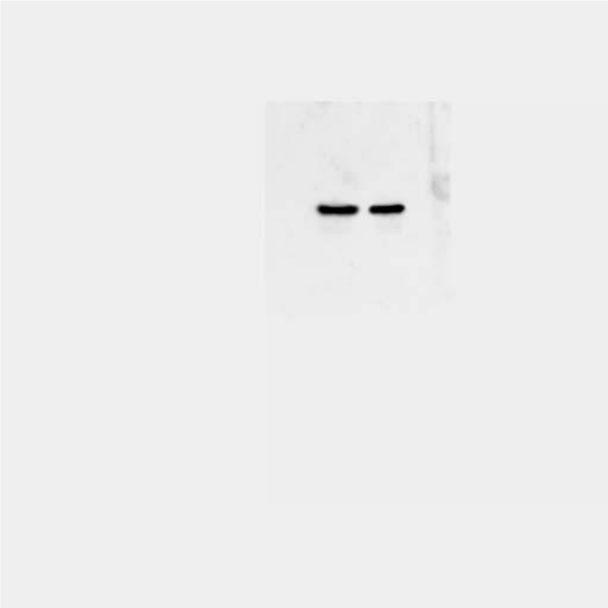

d

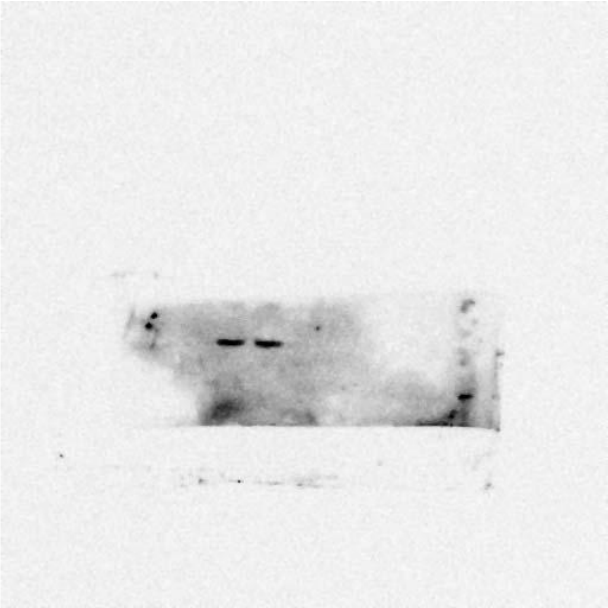

e

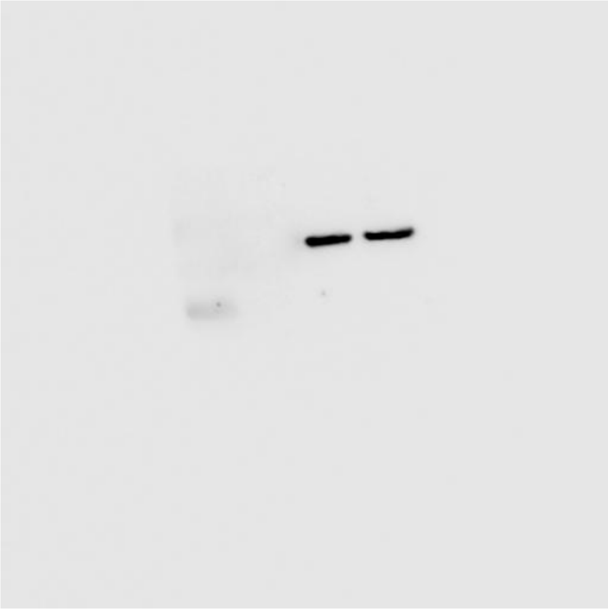

Supplement: Supplementary file 2 — Additional file 2. The raw data of western blot of cyclin D1, cyclin E, CDK4, CDK6 and GAPDH. a cyclin D1 protein levels, (b) cyclin E protein levels, (c) CDK4 protein levels, (d) CDK6 protein levels and (e) GAPDH protein levels were examined by western blot analysis in MC3T3-E1 cells transfected with either control siRNA or Orai1 siRNA. GAPDH was used as an endogenous control. [file 12891_2022_5311_MOESM2_ESM.pdf]
