# Supplementary material for: Orai1 downregulation causes proliferation reduction and cell cycle arrest via inactivation of the Ras-NF-κB signaling pathway in osteoblasts
Source: BMC Musculoskelet Disord. 2022 Apr 11;23:347. doi: 10.1186/s12891-022-05311-y (PMC8996479; doi:10.1186/s12891-022-05311-y)

Uncropped Western blot images

Additional file 3 The raw data of western blot of Ras-GRF, Ras, p-p65-NF-κB, p65-NF-κB and GAPDH. (a) Ras-GRF protein levels, (b) Ras protein levels, (c) p-p65-NF-κB protein levels, (d) p65-NF-κB protein levels (e) GAPDH protein levels were examined by western blot analysis in MC3T3-E1 cells transfected with either control siRNA or Orai1 siRNA. GAPDH was used as an endogenous control.

a

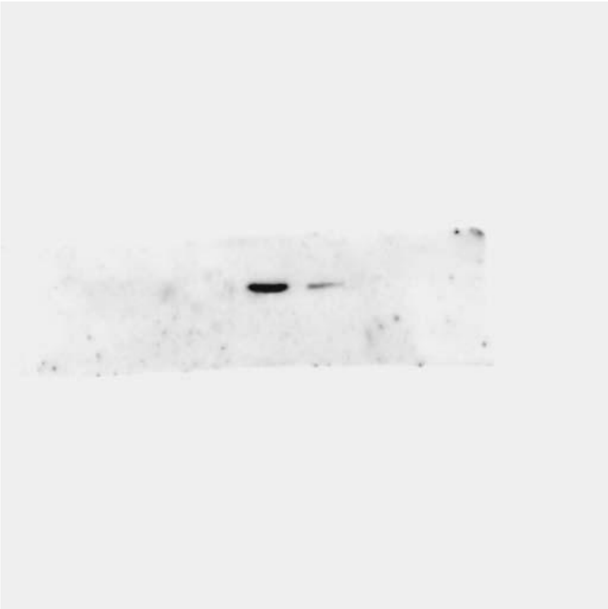

b

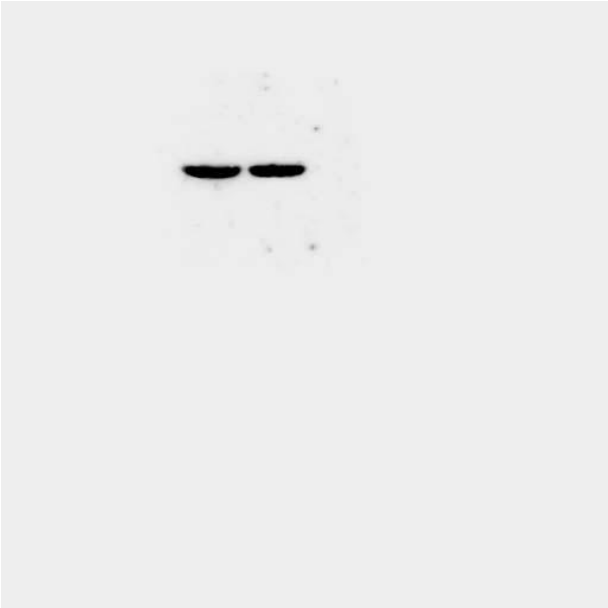

c

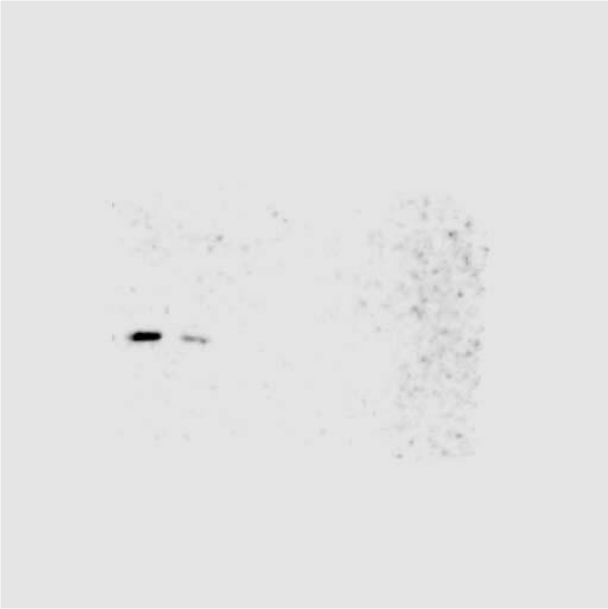

d

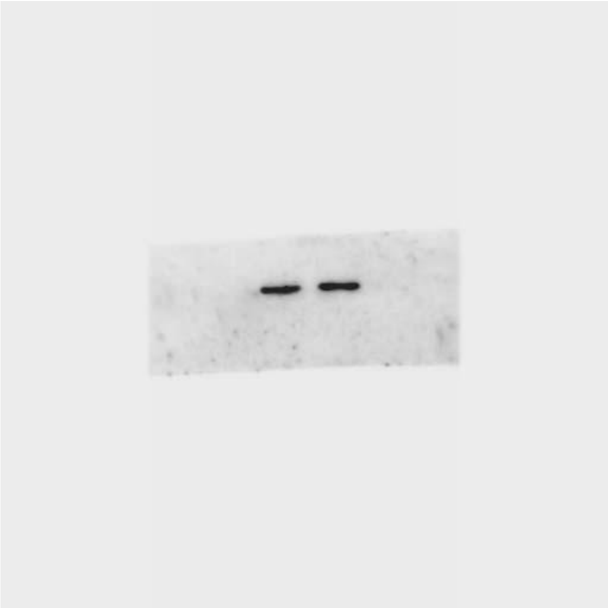

e

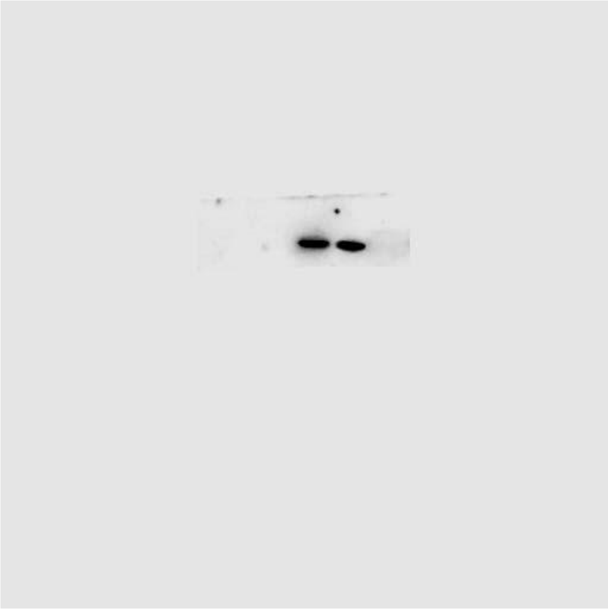

Supplement: Supplementary file 3 — Additional file 3. The raw data of western blot of Ras-GRF, Ras, p-p65-NF-κB, p65-NF-κB and GAPDH. a Ras-GRF protein levels, (b) Ras protein levels, (c) p-p65-NF-κB protein levels, (d) p65-NF-κB protein levels (e) GAPDH protein levels were examined by western blot analysis in MC3T3-E1 cells transfected with either control siRNA or Orai1 siRNA. GAPDH was used as an endogenous control. [file 12891_2022_5311_MOESM3_ESM.pdf]
