# Supplementary material for: Orai1 downregulation causes proliferation reduction and cell cycle arrest via inactivation of the Ras-NF-κB signaling pathway in osteoblasts
Source: BMC Musculoskelet Disord. 2022 Apr 11;23:347. doi: 10.1186/s12891-022-05311-y (PMC8996479; doi:10.1186/s12891-022-05311-y)

Uncropped Western blot images

Additional file 4 The raw data of western blot of p-p65-NF-κB, p65-NF-κB, Cyclin D1 and GAPDH. After incubation with or without ARS-853, the amounts of (a) p-p65-NF-κB protein levels, (b) p65-NF-κB protein levels, (c) cyclin D1 protein levels and (d) GAPDH protein levels were evaluated in the MC3T3-E1 cells transfected with either the control siRNA or Orai1 siRNA. GAPDH was used as an endogenous control.

a

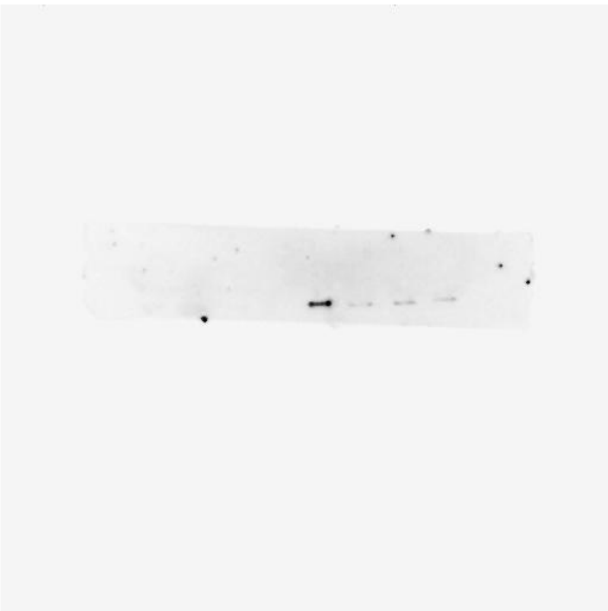

b

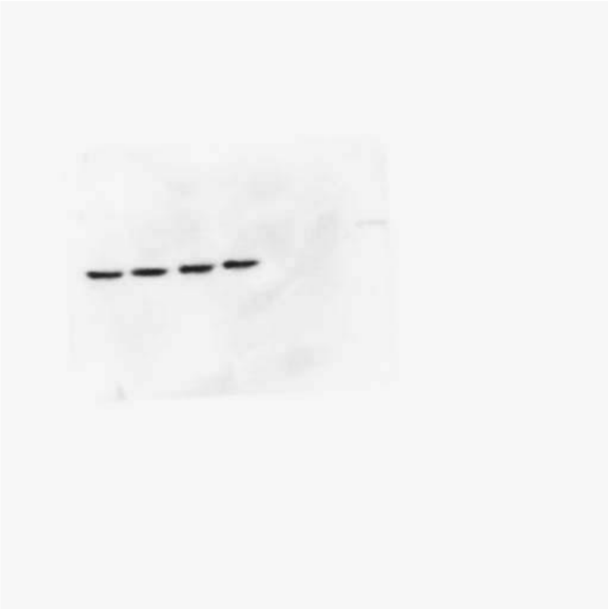

c

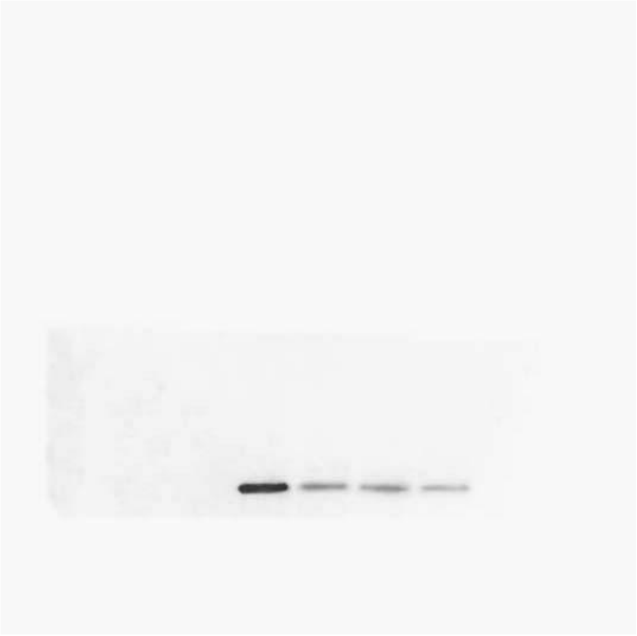

d

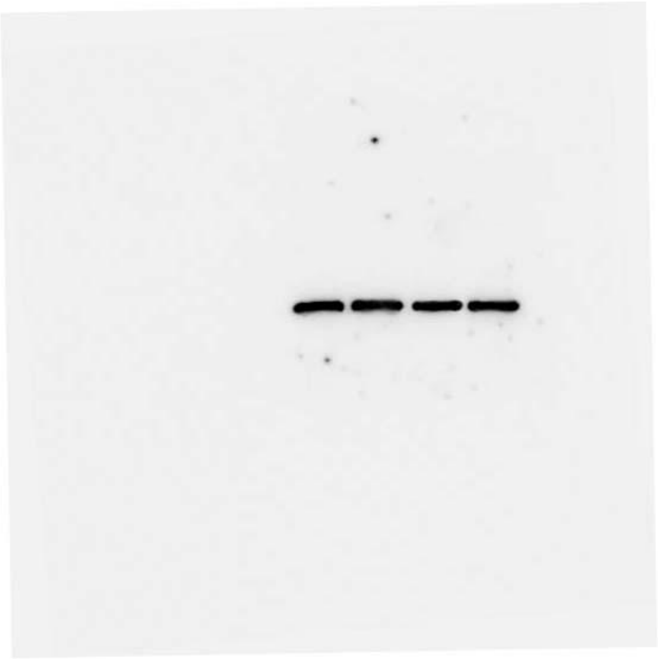

Supplement: Supplementary file 4 — Additional file 4. The raw data of western blot of p-p65-NF-κB, p65-NF-κB, Cyclin D1 and GAPDH. After incubation with or without ARS-853, the amounts of (a) p-p65-NF-κB protein levels, (b) p65-NF-κB protein levels, (c) cyclin D1 protein levels and (d) GAPDH protein levels were evaluated in the MC3T3-E1 cells transfected with either the control siRNA or Orai1 siRNA. GAPDH was used as an endogenous control. [file 12891_2022_5311_MOESM4_ESM.pdf]
